# Single-Molecule FRET Imaging of Virus Spike–Host Interactions

**DOI:** 10.3390/v13020332

**Published:** 2021-02-21

**Authors:** Maolin Lu

**Affiliations:** Department of Microbial Pathogenesis, Yale University School of Medicine, New Haven, CT 06536, USA; maolin.lu@yale.edu

**Keywords:** single-molecule imaging, Förster resonance energy transfer (FRET), virus–host interactions, spike proteins, virus entry, viral membrane fusion, conformational dynamics, SARS-CoV-2, HIV-1, influenza and Ebola

## Abstract

As a major surface glycoprotein of enveloped viruses, the virus spike protein is a primary target for vaccines and anti-viral treatments. Current vaccines aiming at controlling the COVID-19 pandemic are mostly directed against the SARS-CoV-2 spike protein. To promote virus entry and facilitate immune evasion, spikes must be dynamic. Interactions with host receptors and coreceptors trigger a cascade of conformational changes/structural rearrangements in spikes, which bring virus and host membranes in proximity for membrane fusion required for virus entry. Spike-mediated viral membrane fusion is a dynamic, multi-step process, and understanding the structure–function-dynamics paradigm of virus spikes is essential to elucidate viral membrane fusion, with the ultimate goal of interventions. However, our understanding of this process primarily relies on individual structural snapshots of endpoints. How these endpoints are connected in a time-resolved manner, and the order and frequency of conformational events underlying virus entry, remain largely elusive. Single-molecule Förster resonance energy transfer (smFRET) has provided a powerful platform to connect structure–function in motion, revealing dynamic aspects of spikes for several viruses: SARS-CoV-2, HIV-1, influenza, and Ebola. This review focuses on how smFRET imaging has advanced our understanding of virus spikes’ dynamic nature, receptor-binding events, and mechanism of antibody neutralization, thereby informing therapeutic interventions.

## 1. Introduction

**Figure 1:**
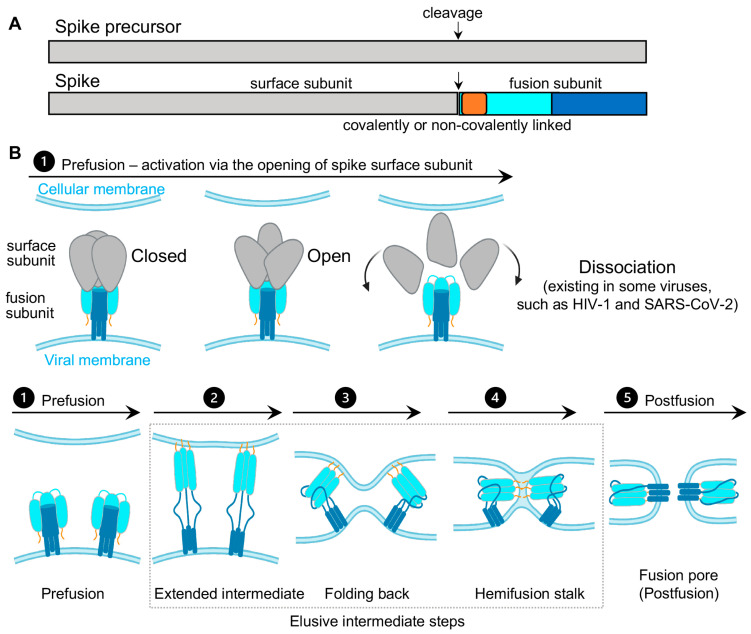
Class I viral fusion proteins and proposed model of viral membrane fusion. (**A**) Schematic drawing of spike precursor and cleaved spike. The spike protein is initially synthesized as a single-chain polypeptide (spike precursor) and later cleaved into a trimer of covalently or non-covalently linked heterodimer. The heterodimer consists of the surface receptor-binding subunit (gray) and the fusion subunit (fusion peptide or fusion loop (FP/FL), dark yellow; N-terminal domain, cyan; C-terminal domain, dark blue). (**B**) Proposed conformational events of virus spikes during viral membrane fusion. These events are as follows, involving conformational changes in the surface subunit (top row) and changes in the fusion subunit (low row, simplified by only showing the fusion subunit [1]). (**1**) Prefusion—conformations of the spike in “closed” and open forms. Spike activation proceeds through an opening of the trimer, usually in response to binding to receptor or due to a cellular cue such as low pH. For non-covalently linked spikes, dissociating/decoupling between the surface/exterior subunit with the fusion subunit has been observed/suggested after the spike opens, such as HIV-1 and SARS-CoV-2 spikes. FP/FL remains sequestered in this process. (**2**) Exposing, extending, and inserting the FP/FL into the cellular membrane leads to the formation of an extended prehairpin intermediate. (**3**) Folding back the C-terminal segment of the fusion subunit back on the N-terminal segment core brings viral and cellular membranes into proximity. (**4**) Further folding and dragging two membranes into contact promotes two membranes’ merging to form a hemifusion stalk. (**5**) The fusion subunit folds into a stable post-fusion conformation, allowing a fusion pore to form. The intermediate steps from (**2**) to (**4**) remain elusive. This proposed model does not specify or speculate the number of spikes required for fusion pore formation.

Virus spikes on the surface of enveloped viruses are often also viral fusion proteins that mediate the fusion between viral membranes and cellular membranes (Figure 1) essential for virus entry [1,2,3]. The merging of virus and lipid bilayers progresses through a hemifusion intermediate, followed by a fusion pore widening, content mixing, and the delivery of virus capsids into the host cytoplasm [4]. Viral fusion proteins respond to the binding of cellular receptors or acidic pH to undergo conformational rearrangements, which eventually promote membrane fusion. Viral fusion proteins have been categorized into three classes [1,2], of which Class I viral fusion proteins include the medically important SARS-CoV-2 spike (S) protein, the HIV-1 envelope (Env) protein, influenza hemagglutinin (HA), and Ebola glycoprotein (GP). These virus spikes are first synthesized as trimers of a single-chain polypeptide—an immature precursor, then go through proteolytical processing by host proteases to form mature spikes—trimers of heterodimers (Figure 1A). Mature spikes are highly metastable on the virus surface. Upon interacting with hosts, mature spikes undergo conformational changes from pre-fusion conformations to the lowest-energy post-fusion conformation (a common hairpin-like or the analogous coiled-coil conformation) through hypothetical intermediates in which the fusion peptide extends and inserts into the host target membrane (Figure 1B). Numerous pre-fusion and post-fusion structures of virus spikes have provided unprecedented details of conformations at individual steps during the viral membrane fusion [5,6,7,8,9,10,11,12,13,14,15,16,17,18,19,20,21]. The recent dynamic studies on virus spikes established platforms to connect these structural snapshots in real time, revealed the order and the kinetics of transitional events, and guided developing interventions aiming to arrest or block viral membrane fusion, thus stopping viral infection [22,23,24,25,26,27,28,29,30,31].

As spikes are highly exposed to our immune system, they are main targets of neutralizing antibodies and thus are critical for developing vaccines and anti-spike therapeutics. Most vaccines or vaccine candidates for HIV-1/AIDS and SARS-CoV-2/COVID-19 are based on their spike proteins to trigger the immune system to produce neutralizing antibodies. Interestingly, in the face of immune pressure, many viral spike proteins use conformational masking of vulnerable antibody-targeted epitopes. In addition to glycan shields and hypermutations, this strategy of conformational masking has been best-understood in HIV-1 [32,33,34,35,36,37]. 

Collectively, virus spike proteins are structurally flexible and conformationally dynamic on the virus surface. The dynamic nature and conformational plasticity of spikes enables entry of enveloped viruses into host target cells through membrane fusion, while distracting the immune system from antibody recognition. Molecular understanding and modulation of spikes’ intrinsic dynamics can guide the rational design of spike-targeting interventions such as vaccines, small-molecule inhibitors, and antibodies to block virus entry. Single-molecule Förster resonance energy transfer (smFRET), a spectroscopic tool sensitive to inter-fluorophore molecular distances, has been well-positioned to capture the innate dynamics of virus spikes labeled with Förster resonance energy transfer (FRET)-paired fluorophores. This minireview covers recent work on single-molecule FRET imaging of class I fusion proteins of enveloped viruses, including SARS-CoV-2 [31], HIV-1 [22,23,24,25,26,27], influenza [29], and Ebola [28,30]. 

## 2. Single-Molecule Förster Resonance Energy Transfer (smFRET) Imaging

Imaging macromolecules at the single-molecule/single-particle level has advanced our understanding of both static and dynamic aspects in virus–host interactions, merited by avoiding the averaging-out effect from traditional ensemble-level measurements. Those imaging techniques, such as single-particle cryoEM/cryoET, single-particle optical tracking, and super-resolution fluorescence microscopy exerted significant roles in addressing fundamental questions with regards to structures, dynamics, and functions of virus molecules underlying virus–host interactions [38,39,40,41,42,43,44]. 

Single-molecule Förster resonance energy transfer (smFRET) imaging has been proven to be a reliable imaging tool for revealing the intrinsically dynamic, heterogeneous nature of biological systems [45,46,47]. It is powerful to probe protein conformational dynamics in a time-resolved manner; identify previously unknown states; detect intermediates transient in nature; and delineate the sequence, the order, the timing, the frequency of conformational states, and the transitional events. Two significant advantages of smFRET are (1) it permits direct in situ observations of different conformations/structures of biological molecules in real time, and (2) it reveals conformational intermediates and features that are previously concealed or averaged-out in ensembles. 

**Figure 2:**
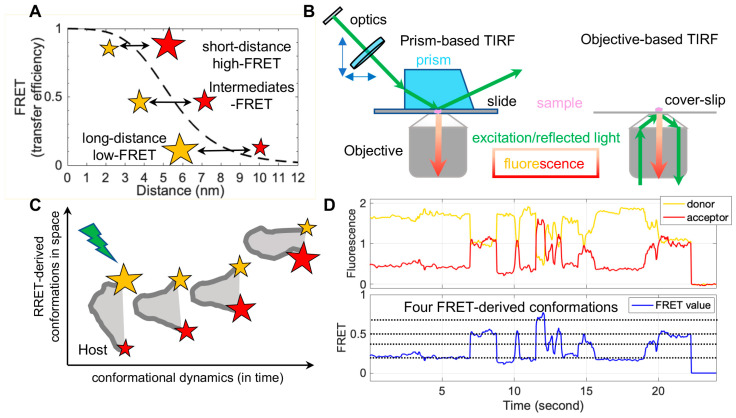
Single-molecule Förster resonance energy transfer (smFRET) principle, instrumentation, and imaging of dynamic biomolecules. (**A**) An example curve depicting energy transfer efficiency (Förster resonance energy transfer (FRET), dashed black line) from an excited donor fluorophore to a neighboring acceptor fluorophore as a function of donor–acceptor distances. FRET values or FRET negatively correlate with the distance within a couple of nanometers between a donor (yellow star) and an acceptor (red star). (**B**) Widely used smFRET imaging instrumentations: prism- and objective-based total internal reflection fluorescence (TIRF). (**C**,**D**) Real-time observations of conformational motions in biomolecules by smFRET. (**C**) Diagram depicting ideal FRET-derived space-time coordinates of biomolecule conformations. Donor, yellow star; acceptor, red star; relative fluorescence intensity, the star’s size; biomolecule of interest, gray. (**D**) Example FRET-related traces showing four interconvertible conformations in real time. The host molecule dynamically samples four conformations, reflected by different donor–acceptor energy transfer efficiencies. Donor fluorescence trace, solid yellow line; acceptor fluorescence trace, solid red line; calculated FRET trace, solid blue line; FRET-indicated conformations, dashed black lines.

FRET refers to non-radiative energy transfer between a donor and acceptor fluorophore. The energy transfer efficiency is a function of distances between both fluorophores, described as FRET = 1/(1 + (*R*/*R*_0_)^6^), where *R* is the inter-fluorophore distance and *R_0_* is the Förster distance determining the range of sensitive measure of distance [47]. For mostly used paired-fluorophores, the FRET-detectable distance ranges from 30 Å to 80 Å, well suited for the dimensions of spike proteins of enveloped viruses (Figure 2A). In application, a pair of donor and acceptor fluorophores is site-specifically labeled on the one or two molecules of interest. The donor fluorophores are excited by a laser, and the single-molecule fluorescence from both fluorophores is separately recorded for seconds to minutes by total internal fluorescence microscope (TIRF) or confocal microscopy [47], in which prism-based TIRF and objective TIRF are widely used (Figure 2B). Fluorescence and the quantified FRET values monitor the proximity between two fluorophores in real-time, ultimately translating to the object’s intra-molecular or inter-molecular dynamics (Figure 2C,D). The object of interest can be genetic materials, proteins, peptides, and other biomolecules. The applications of FRET in biological systems are broad, thanks to advances in instrumentations, analysis software, and site-specific dye-labeling methods. For instance, newly developed scientific CMOS (sCMOS) cameras and the developed smFRET software or algorisms [48,49,50,51] facilitate high-throughput smFRET imaging and robust data analysis.

In contrast, the attachment of fluorophores to specific sites on proteins without disturbing their functionality has been a technical obstacle. The conventional way to label proteins is to introduce cysteines, which permit maleimide-functionalized dyes’ attachments [52]. A considerable number of pre-existing essential cysteines on virus proteins, especially spike proteins, make it infeasible. Two alternative fluorophore-attaching strategies, enzymatic and amber-click labeling, have overcome this hurdle. Enzymatic methods take advantage of enzymes that site-specifically recognize short-peptide tags (6–12 amino acids in length) introduced into the protein of interest and transfer dye-conjugated substrates to these tags [53,54]. The genetically encoded copper-free click chemistry (amber-click) allows reading through introduced amber stop codons on the protein of interest as unnatural amino acids through amber suppression, followed by site-specifically labeling conjugated dyes on unnatural amino acids by copper-free click chemistry [55,56]. Both enzymatic and amber-click methods have been applied to study virus spikes for many enveloped viruses to reveal dynamic aspects during viral membrane fusion [22,23,24,25,26,27,28,29,30,31]. 

## 3. Conformational Dynamics of Virus Spike Proteins on the Surface of Viruses

### 3.1. Conformational Modulations of SARS-CoV-2 Spikes by Receptor and Antibodies

**Figure 3:**
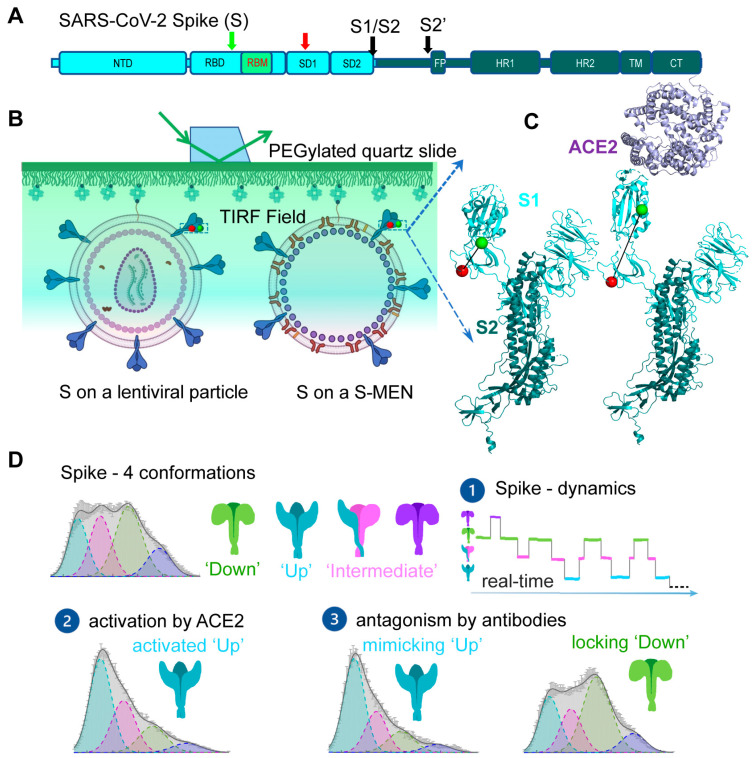
Conformational modulations of dynamic SARS-CoV-2 spike (S) proteins by receptors and antibodies. (**A**) Domain organization of full-length wild-type SARS-CoV-2 S (S1, cyan; S2, dark blue). Green and red arrows indicate the Cy3- and Cy5-labeling sites, respectively. Black arrows indicate protease cleavage sites (S1/S2 and S2′). NTD, N-terminal domain; RBD, receptor-binding domain; RBM, receptor-binding motif; SD1, subunit domain 1; SD2, subunit domain 2; FP, fusion peptide; HR1/HR2, heptad repeat 1/heptad repeat 2; TM, transmembrane domain; CT, cytoplasmic tail. (**B**,**C**) smFRET imaging experimental set-up. (**B**) Virus particles carrying a fluorescently labeled SARS-CoV-2 S protomer among wild-type spikes were immobilized and imaged on a PEG/PEG–biotin-coated PEGylated quartz slide on a prism-based TIRF microscope. The same type of experimental strategy has been used in other virus spike proteins throughout this review. For SARS-CoV-2, two virus particle systems were used to carry S on the surface. HIV-1 lentivirus particles are composed of HIV-1 cores and S proteins on the surface. S-MEN comprises four structural proteins of SARS-CoV-2 (S, spike; M, membrane protein; E, envelope protein; and N, nucleocapsid protein. (**C**) The binding of the cellular receptor human angiotensin-converting enzyme 2 (hACE2) induces conformational changes of S from the “RBD-down” (based on PDB:6VSB) to the “RBD-up” (PDB: 6VYB/6M0J) conformation. Cy3-labeling site, green ball; Cy5-labeling site, red ball; S1, light cyan; S2, dark blue; hACE2, magenta. (**D**) Featured findings of S conformations by smFRET imaging. These findings include (1) S dynamically samples four different conformations in real time, and S is in equilibrium exchange between states; (2) the binding of receptor hACE2 shifts S from the ground state (“RBD-down”) to the activated state (“RBD-up”) via an on-path intermediate (existing in the asymmetric S); (3) antibodies can antagonize S either by directly competing with receptor for the binding to S or by stabilizing S in the ground state (“RBD-down”).

During the current COVID-19 pandemic, smFRET was applied to study SARS-CoV-2 viral spike (S) glycoprotein and reveal real-time conformational dynamics of S [31]. S mediates the entry of SARS-CoV-2 into host cells through membrane fusion and is the primary target for antibody responses; thus, it is an attractive target for COVID-19 vaccines and treatments [57,58,59,60,61]. S is initially synthesized as a single-chain polyprotein precursor and is proteolytically cleaved by furin protease into a trimer of non-covalently linked S1/S2 heterodimer, followed by a second cleavage by cellular proteases such as TMPRSS2 or cathepsin B to generate S’ (Figure 3A) [62,63]. The receptor-binding domain (RBD) of S recognizes human angiotensin-converting enzyme 2 (hACE2) on human cells. This receptor-binding event initiates conformational changes in S that promote viral membrane fusion to allow virus entry [64,65]. Structures of S in both soluble form and virus-associated form have been characterized at high-resolution, revealing atomic or near-atomic details of different S conformations [5,6,7,66,67,68,69,70,71,72,73,74,75]. Major prefusion conformations include a closed trimer with all RBD oriented “down”, occluding the binding site for hACE2, and open trimers with at least one RBD oriented “up”, accessible to hACE2 [5,6,7,66,67,68,69,70,71,72,73,74,75]. Real-time information connecting these structural snapshots and additional intermediates has been obtained by smFRET (Figure 3) [31]. 

smFRET imaging of SARS-CoV-2 spike protein [31] has revealed conformational transitions from a closed ground state to the open receptor-stabilized conformation via an on-path intermediate. SARS-CoV-2 spikes were incorporated into the surface of lentivirus- and coronavirus-like particles. Two dyes were enzymatically attached to introduced short peptide labeling tags on S1 without compromising S functionalities. Real-time monitoring of individual S carrying one pair of FRET dyes in the context of viral particles showed that S is dynamic on the virus surface (Figure 3B,C). It sampled an ensemble of the four most populated conformational states, reflected by distinct levels of FRET values: low FRET (0.1), intermediate FRET (0.3), intermediate FRET (0.5), and high FRET (0.8) (Figure 3D). The intermediate FRET (0.5) state was identified as the ground state—a closed spike, in which three RBDs are oriented “down” toward viral membranes. Spikes on the surface of lentivirus- and coronavirus-like particles predominately reside in this state. In the closed spike, a stabilized spike mutant with a disulfide bridge between position S383C and D985C further enriched the occupancy of closed spikes, consistent with findings from EM studies [67]. The low FRET (0.1) was identified as the activated “open” spike where RBD is oriented “up” towards the receptor hACE2. Direct observations of individual spikes on the virus over time and accumulated state-population histograms revealed an on-path intermediate during the spike opening from RBD “down” to RBD “up” (Figure 3D). The estimation of site-to-site distances between two labeling tags implied that the on-path intermediate likely originates from one or two neighboring hACE2-free protomers to the hACE2-bound “up” protomer within an asymmetric S configuration (one-RBD-up or two-RBD-up) [75]. The remaining high-FRET (0.8) remains an unassigned conformation. smFRET imaging of conformations of S was in global agreement with extant high-resolution structures [5,6,7,66,67,68,69,70,71,72,73,74,75], suggesting that the spike undergoes sequential activations of three protomers from symmetric three-RBD-down, asymmetric one-RBD-up, or two-RBD-up, to symmetric three-RBD-up configurations. smFRET also showed that proteolytic processing of S by the TMPRSS2 [62,63,76], mimicking serine protease trypsin, enhanced hACE2-dependent activation of S, which was confirmed by virus–cell and cell–cell fusion assays [31,77].

One unique strength of smFRET is to reveal the timing, order, and frequency of conformational states and state-to-state transitions of S in situ on the surface of viral particles (Figure 3D) [40,41,47]. Kinetic analyses deployed in smFRET include the transition density for state-to-state transition (plotted as initial state vs. final state), the hidden Markov modeling for idealizing molecular motions, and dwelling time for estimating transitional rates. Results from these analyses revealed dynamic aspects of S: (1) connecting extant high-resolution structural snapshots in time with the scale ranging from milliseconds to seconds, (2) S exhibiting a defined transition order between four distinct conformations, (3) ligand-free S being in a dynamic equilibrium of four different states, (4) host receptor hACE2 re-equilibrating the balance by accelerating transitions into the activated state, and (5) relative free energy landscape derived from dynamics providing a qualitative sense for the activation of S [31].

smFRET analysis of S also allowed an assessment of neutralizing monoclonal antibodies [7,70,78,79] and convalescent patient plasma [80,81]. Two different neutralization features were identified, one preferring the open S, the hACE2-bound RBD “up” conformation, whereas others stabilized the close S with RBD “down” conformation. These results suggested two different virus-neutralizing strategies by antibodies: either by direct competition for the binding to ACE2 or via stabilizing S into the “down” conformation (Figure 3D). The observation of the allosteric mechanism from smFRET imaging appears to be in line with the success of the current three COVID-19 authorized vaccines, namely, Moderna mRNA, Pfizer mRNA, and AstraZeneca [57,59,60]. All three approved vaccines encode for different forms of S that predominantly present the “down” conformation. Scientific observations from structural studies and smFRET imaging provide the basis for interpreting the host response to vaccines. 

By the time of writing this review, more spike (S)-based SARS-CoV-2 vaccines have shown promising efficacy in inducing neutralizing antibodies to either completely protect or dramatically reduce severe illnesses. On the other hand, new S variants are emerging across the globe, such as in the United Kingdom B.1.1.7 (eight mutations in S), South African B.1.351 (eight mutations in S), and Brazilian B.1.1.248 (12 modifications in S). In early 2020, the D614 strain quickly displaced the original G614 (Wuhan strain) and became the dominant pandemic form in many countries [82,83]. Understanding whether emerging S variants will influence current vaccines’ effectiveness becomes essential for strategizing to curb the pandemic. As these and future alternations in S can potentially affect the effectiveness of current vaccines, vaccine boosts for new S variants might be required. The development of effective anti-viral drugs is also in urgent demand. Both S1 and S2 subunits are attractive druggable targets. The subunit S2 as the actual fusion machine is highly conserved. Despite their importance, direct observations of S2 conformational changes associated with fusion have been lacking. How does S1 decouple from S2 to allow S2 to proceed towards fusion? Is the proteolytical cleavage to generate S2′ associated with increased FP exposure? What are the structural intermediates between prefusion and postfusion conformations? Insights into long-lived structural intermediates would open the door for drug development targeting S.

### 3.2. Dynamic Aspects of HIV-1 Virus Spike—Env: Conformational States and Implications for Vaccine and Drug Design 

**Figure 4:**
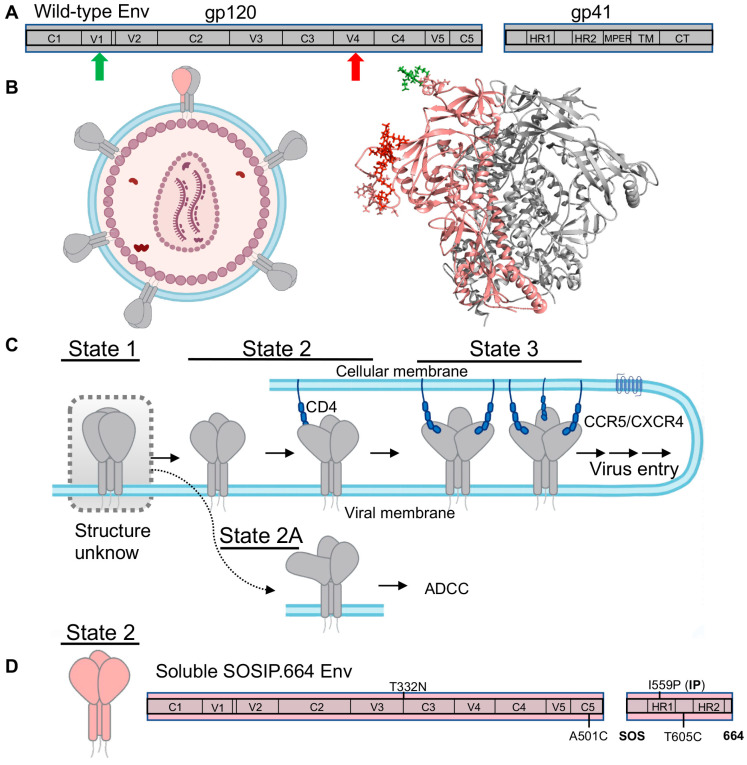
On-path pre-fusion HIV-1 envelope (Env) trimer conformations identified by smFRET imaging. (**A**) Scheme of wild-type HIV-1 Env, noncovalently associated gp120 and gp41 subunits. (**B**) smFRET imaged individual HIV-1 viruses carrying a fluorescently labeled protomer within an Env trimer and elsewhere wild-type trimers. The Cy3/Cy5-labeled protomer (Cy3, green; Cy5, red) is in pink, whereas other wild-type protomers are colored gray. Structure is made based on PDB accessions 4ZMJ and 5FUU. (**C**) Model of Env activation by sequential binding of CD4 receptors. Env dynamically samples three primary conformational states in which State 1 is the predominant one. Upon sequential activation by CD4, Env transits through an asymmetric State 2 to a completely open State 3 (two- or three-CD4-bound trimer). State 2-Env is a single CD4-bound asymmetric trimer, in which the CD4-bound protomer adopts State 3 and the neighboring free protomers adopt State 2. State 2A is an off-path conformation that is highly vulnerable to antibody-dependent cellular cytotoxicity (ADCC). Following CD4 activation, the binding of coreceptors CCR5/CXCR4 lead to virus entry, which smFRET has not informed. (**D**) Vaccine candidates based upon soluble SOSIP.664 Env trimer resemble State 2, consisting of three State 2 protomers. The design of soluble SOSIP.664 Env is illustrated in the schematic. Results from (**D**) infer that State 1 Env (**C**) is structurally unknown.

HIV-1 surface envelope (Env) glycoprotein, as the only protein outside the virus, is of critical importance for developing HIV-1 vaccines and anti-Env drugs. Env is initially synthesized and trimerized into Env precursor, a trimer of gp160, and then cleaved by a host furin-like protease into mature Env, a trimer of non-covalently associated gp120/gp41 heterodimers (Figure 4A,B) [84]. The gp120 is the exterior subunit and the gp41 is the transmembrane subunit [84]. Upon engaging with cellular receptor CD4 and coreceptors (CCR5 or CXCR4), HIV-1 Env undergoes a series of conformational changes or structural rearrangements in both subunits—the receptor CD4-binding event induces conformational changes in gp120 to expose coreceptor-binding sites. Subsequent CCR5 or CXCR4 binding to gp120 is believed to activate a cascade of refolding events in gp41, eventually leading to the formation of a six-helix bundle that eventually leads to viral membrane fusion [8,9,10,11,12,32,84,85,86,87,88,89,90,91,92,93,94]. 

Env is also the target for neutralizing antibodies. In order to bind receptor and co-receptor, Env must open. However, these open Env conformations are extremely vulnerable to antibody recognition. As a consequence, HIV-1 Env changes conformations to conceal receptor and coreceptor binding sites by closing, and the easily elicited initial antibodies become non-neutralizing against the closed trimer [33,37,95,96]. These close Env conformations are very difficult to be recognized by antibodies. It often takes years of antibody maturation and Env-antibody co-evolution for antibodies to arise and neutralize these closed conformations [37,97,98,99,100]. Interestingly, the emerging antibodies are often broadly neutralizing antibodies (bnAbs), meaning that they can recognize Env trimer features conserved across most HIV-1 isolates. These bnAbs target conserved regions or epitopes on Env, including the CD4 binding site, V1/V2-glycan site, V3-glycan site, MPER (membrane-proximal external region), and fusion peptide domain [96,97,101,102,103,104,105,106]. Thus, Env is the research focus for developing HIV-1 interventions, such as Env-mimicking vaccines aiming for inducing bnAbs, antibody therapy with bnAbs, and drug treatment with CD4-mimics or fusion inhibitors. 

smFRET has permitted access to dynamic aspects and conformational profiles of HIV-1 Env in the context of intact virions [22,23,24,25,26,27]. Both enzymatic labeling and amber-click labeling have been used to label HIV-1 Env site-specifically with fluorophores. The intact HIV-1 virion, in which only a protomer within an Env trimer is fluorescently labeled while other Envs remain wild-type (Figure 4B), was immobilized and imaged on a prism-TIRF microscope. smFRET revealed that the unliganded HIV-1 Env on the virus is intrinsically dynamic in real time, and it continuously transits between at least three conformational states, the so-called State 1, State 2, and State 3 (Figure 4C) [22,23,107]. Efforts on identifying the three states’ nature led to the delineation of a stepwise Env activation by receptor CD4 molecules. During the activation process, Env on the surface of viruses initially primarily resides in the pre-triggered conformation (State 1, most-closed), gradually transits through a default conformation (State 2) in which a single CD4 is engaged with the trimer, and eventually arrives at the completely open conformation (State 3) with three CD4 molecules bound (Figure 4C) [23]. A recent collaborative study of smFRET, cryo-electron tomography (Cryo-ET), and antibody-binding assay parallelly uncovered an additional asymmetric Env conformation, State 2A (Figure 4C), as a fourth conformation identified for Env, which is connected to State 2 [24]. State 2A is highly vulnerable to antibody-dependent cellular cytotoxicity (ADCC). In ADCC, Env bound with non-neutralizing or poorly neutralizing antibodies (CD4-induced anti-cluster A antibodies, coreceptor binding antibody 17b) displayed on the surface of infected cells can be recognized by Fc-receptor expressing cytotoxic CD8+ T cells or natural killer cells [108]. This finding suggests that ADCC likely plays an important role in clearing the more open Env conformations, which can be exploited therapeutically by forcing Env into open conformations using CD4 mimetics [24,109]. 

Moreover, smFRET imaging has provided mechanistic understandings of how Env trimer responds to anti-viral inhibitors [22,25,27]. One of the significant findings is the identification of one very potent entry inhibitor BMS-626529/temsavir. A prodrug version Fostemsavir is now an FDA-approved drug in the United States for highly treatment-experienced patients with multidrug-resistant HIV-1 infection who are failing their current antiretroviral therapy regimen [110,111]. smFRET shows that temsavir is able to reduce the occupancy of downstream open conformations and stabilize Env into the closed State 1 [22,25,27]. This drug inhibits Env by blocking its activation towards trimer opening on the fusion path. Designed Env variants with the disrupted allosteric network have been shown to be less sensitive to CD4 activation and resistant to being opened by potent dodecameric CD4 [112]. Thus, the State 1 revealed in smFRET imaging is a tempting target for effective fusion inhibitors. In contrast, small molecule CD4 mimetics are shown to stabilize the open conformation State 3 [22]. CD4 mimetics likely antagonize Env by prematurely activating Env or driving Env off-pathway.

HIV-1 Env induces human immune responses to produce Env-targeting antibodies. Which conformation of Env that neutralizing and non-neutralizing antibodies preferentially bind is critical for designing Env-mimicking vaccines with the aims of inducing bnAbs but not non-neutralizing antibodies. By testing bnAbs directed against different epitopes on Env, smFRET data show that many bnAbs prefer to recognize the most closed State 1 [22,25,101]. Those antibodies include CD4 binding site-directing bnAbs (VRC01 and 3BNC117), V1V2 glycan site-targeting bnAbs (PG9, PG16, PGT145), and V3-glycan patch bnAbs (10–1074, PGT128, PGT122) [22,25,101]. In contrast, non-neutralizing antibodies (17b, F105) stabilize the open State 3, indicating that State 3 Env induces non-neutralizing antibodies [22,25]. Notably, smFRET also demonstrates that the Env precursor (gp160)_3_ on the intact virus exhibits more open conformations of State 2 and State 3 in contrast to the mature Env [27]. A large number of Env precursor displayed on the surface of cells is likely a strategy used by HIV-1 to distract the host immune system from the induction of State 1-targeting neutralizing antibodies through presenting epitopes for non- or poorly neutralizing antibodies [113,114]. Most importantly, the fact that many bnNAbs and fusion inhibitors preferentially recognize prefusion State 1 Env has significant implications for vaccine development. The preference towards State 1 implies that State 1 is most likely effective for triggering immune responses to elicit bnAbs. Therefore, Env-mimicking vaccine candidates designed to mimic the primary conformation of Env and aimed at inducing bnAbs should present the conformation that many bnAbs have preferences for the State 1 Env. 

One of the most promising Env-based HIV-1 vaccine candidates is called SOSIP.664, and is derived from the BG505 subtype. SOSIP.664 is a recombinant, soluble, truncated gp140 trimer, engineered by introducing a disulfide bond between the gp120 and gp41 (SOS), an I559P change in gp41 (IP), and truncation at residue 664 (Figure 4D) [91,115,116]. These modifications make highly dynamic Env relatively stable, which significantly advances vaccine studies and has allowed a breakthrough in the structural characterizations of trimeric Env at the atomic level [11,12,91]. Subsequently, it resulted in high-resolution structures of trimeric Envs in ligand-free, antibody-bound, or small molecule-bound forms [11,12,93,101,117,118,119,120,121]. Interestingly, except the CD4-bound open Env trimer [93,120,121], most pre-fusion Env structures exhibit a similar architecture, which was assumed to resemble the primary conformation of virus-embedded native Env—State 1 Env. To address whether SOSIP mimics native Env on the viruses, researchers performed smFRET imaging on both BG505 SOSIP.664 and virus wild-type BG505 Env with FRET pair dyes placed at the same positions [25]. Unexpectedly, smFRET revealed that soluble SOSIP.664 resembled the default intermediate, State 2, not the State 1 that virus Env predominantly resided on the virus surface (Figure 4D). This finding suggests that vaccine candidates based on SOSIP.664 designs target the State 2 Env, not the primary target for bnAbs—State 1 Env. This surprising finding agrees with previous concerns in which functional dissimilarities were observed between SOSIP.664 and the physiologic Env on the viral or cellular surfaces [122,123,124]. The finding is also consistent with observed discrepancies between SOSIP.664 and native Env in glycosylation and cross-linking assay profiled by mass spectroscopy [125,126,127].

Scientific observations that support the conclusion of vaccine candidate SOSIP resembling State 2 Env are from several lines of evidence [25]: (1) smFRET reveals that the SOS modification in Env is mostly responsible for the conformational shift of SOSIP.664 towards State 2; (2) multi-dimensional static and dynamic observations of native Env on the viruses validated State 1 Env dominant in ligand-free virus Env; (3) conformational preferences for States 1 and 2 observed in smFRET can be detected using conventional bulk measurement ELISA and flow cytometry; (4) SOSIP-elicited antibodies exhibit a preference for State 2, and the preference is independent of epitopes (CD4 binding site, glycan hole, fusion peptide) and hosts (cows, rabbits, guinea pigs). The finding of SOSIP.664 representing State 2 Env overturned the original assumption of SOSIP.664 being in State 1 and may offer an explanation as to why it is so difficult to generate a vaccine against HIV-1 [25]. The structure of the conformational state behind the State 1 observed by smFRET remains unknown [25]. 

Association of structures to states observed by smFRET likely requires parallel cryoET and smFRET. A first study on chemically inactivated BaL HIV-1 viruses lead to the discovery that the treatment with aldrithiol-2 (AT-2) used to chemically inactivated HIV-1 viruses caused a stabilization of State 2 [26]. cryoET analysis of AT-2-treated Bal viruses generated density maps of Bal Env with much better resolution (below 1 nm) [26], in contrast to previously reported resolution of 2–3 nm [87]. Individual gp120 and gp41 subunits were resolved, and secondary structure densities appeared to be visible [26]. However, State 2 SOSIP structures largely agreed with the obtained density map of Bal Env on the surface of viral particles [26], meaning that structures characterized by cryoET were not State 1 Env identified by smFRET. The subsequent smFRET imaging of several different virus Env subtypes showed that the cross-linking oxidizing reagent AT-2, generally believed to disrupt disulfide bridges in finger motif of retroviral particle nucleocapsid, caused conformational shifts of Env from State 1 to State 2 [26]. The addition of bnAbs could not reverse the shifts caused by AT-2. The detailed mechanism remains unclear, given that a single Env carries ≈60 pre-existing cysteines. To explore and characterize the State 1 Env, researchers should attempt alternative virus-inactivating methods (such as mild oxidizing reagents N-ethylmaleimide or β-propiolactone) for EM imaging purposes. 

Current smFRET analysis of HIV-1 Env is mainly on the receptor-binding subunit gp120. These studies have revealed a fully sequential activation of HIV-1 gp120 by receptor CD4 binding, provided mechanistic understandings of antibody recognition, anti-Env drug function, and Env variants’ immunogenetic functions, and informed on vaccine and therapeutics developments. Nevertheless, there are many remaining questions regarding the mechanistic details of HIV-1 Env-mediated virus–host interactions. For instance, how different is the predicted prefusion State 1 Env from the current State 2 Env? How many trimers on the HIV-1 surface can lead to successful virus entry? What are the dynamics and conformations of gp41 associated with the uncoupling from gp120? What are the conformational events of gp41 during viral membrane fusion? What are the structure and dynamics of the hypothetical pre-hairpin gp41 intermediate? Do membrane compartments and other structured proteins of HIV-1 play roles in membrane fusion? What are the conformational events of the fusion peptide? Answering these questions will guide Env-based vaccines and drug developments. 

### 3.3. Reversible and Irreversible Conformational Events of Influenza A Hemagglutinin Fusion Glycoprotein during Membrane Fusion Process

**Figure 5:**
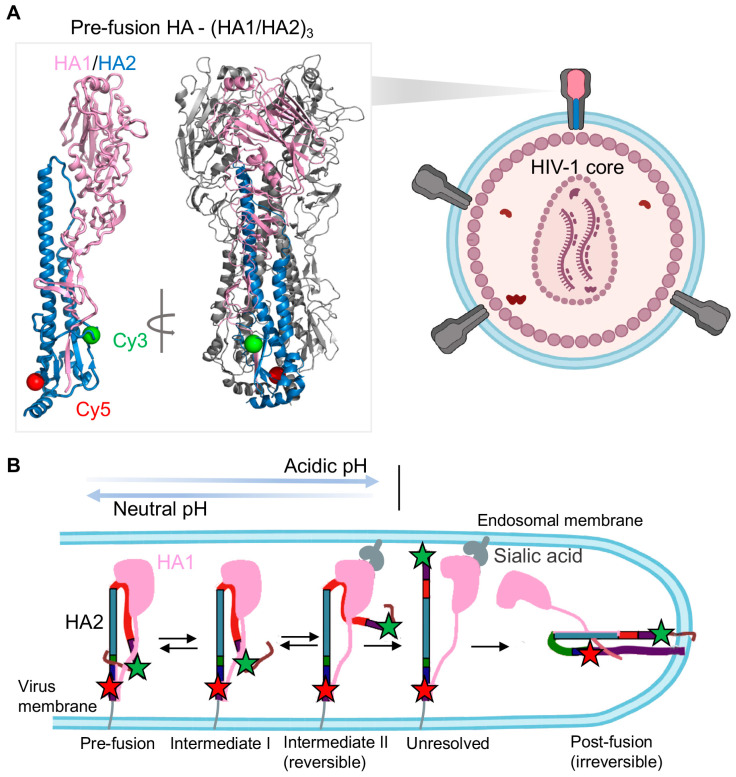
Conformational dynamics of influenza A hemagglutinin (HA) revealed by smFRET imaging. (**A**) Imaging context of HA trimer on viral particles. Lentiviral particles incorporated with HA trimers are imaged at the single-molecule level. On the virus surface, only one HA protomer (color-coded) within an HA trimer is fluorescently labeled. Two fluorophores (Cy3 in green, Cy5 in red) are attached on HA2 at indicated positions. The structure of the pre-fusion HA, the trimer of HA1/HA2 with labeling sites, is shown (on the basis of PDB 2FK0). (**B**) The proposed model depicts reversibly and irreversibly conformational dynamics of HA2 during viral membrane fusion (adapted from [29]). In response to acidic pH and receptors, HA2 shifts conformations from the pre-fusion state to the coiled-coil post-fusion state through multiple fusion-related intermediate states. In the absence of sialic acid receptors, acidic pH triggers HA2 conformational changes in favor of intermediate I and intermediate II, which can be reversed by re-neutralizing the pH. The intermediate I is the conformation in which the fusion peptide is exposed out of the hydrophobic pocket, whereas the fusion peptide in intermediate II is released. The interaction of HA1 to sialic acid-containing endosomal membrane promotes the irreversible process of HA2 adopting coiled-coil post-fusion conformation.

Influenza A hemagglutinin (HA) is one of most studied class I fusion proteins and has served as the model system to characterize viral membrane fusion for many viruses. Recently smFRET imaging has allowed access to the visualization of real-time conformational dynamics of HA (Figure 5) [29]. HA promotes the fusion of the virus with endosomal membrane, allowing virus entry into host cells. The mature HA is structured as a trimer of disulfide-linked HA1/HA2 heterodimers in which surface subunit HA1 contains the receptor sialic acid-binding domain and membrane anchor subunit HA2 carries the fusion domain (Figure 5A). Unlike HIV-1 Env, conformational changes of influenza A “spring-loaded” fusion subunit HA2 is activated by acidification of the endosome, not by the receptor/co-receptor binding; the viral membrane fusion process of influenza A is triggered by low pH [128,129]. The current working model of HA-mediated viral membrane fusion [1,2], which is largely replying on existing HA structures [13,14,15,16,130], suggests two major conformational events in HA2. The first loop-to-helix transition in HA2 induced by low pH exposes the fusion peptide, followed by its extension and insertion into the target membrane. Subsequently, the second helix-to-loop transition in HA2 leads to a coiled-coil formation, the post-fusion conformation. 

The most recent smFRET work of HA2 connected fusion-active states in time, extending our mechanistic understanding of how HA mediates membrane fusion [29]. Das et al. [29] used the amber-click labeling method to introduce two fluorophores in HA2 before and after the conformational switching region. They incorporated dye-labeled HA along with excess wild-type HA proteins on the surface of a lentiviral particle (Figure 5A) and monitored how HA2 conformations responded to pH changes and sialic acid-binding to HA1 (Figure 5B). At neutral pH, HA2 was shown to maintain a dynamic equilibrium among three FRET-indicated populated conformational states: high-FRET, intermediate FRET, and low-FRET states. Lowering pH gradually from neutral to acidic conditions caused gradual shifts of the HA2 conformational landscape from high-FRET dominance to low-FRET dominance through a transient intermediate. They assigned the transient intermediate to a fusion peptide-exposed intermediate in which the fusion peptide was released out of the hydrophobic pocket after being exposed to the acidic environment.

Remarkably, long and transient exposure to acidic pH exert different conformational consequences on HA2, which uncover two different conformations behind the low FRET. Long exposure irreversibly shifted conformations of HA2 to the low-FRET dominance, believed to be the irreversible coiled-coil configuration—the post-fusion conformation. In the presence of receptor sialic acids containing liposomes, HA2 appeared to adopt the coiled-coil conformation even at neutral pH and rapidly transited to that conformation at low pH irreversibly. The role of accelerating the fusion process of receptors agreed with the rapid formation of the coiled-coil post-fusion conformation. On the other hand, transient exposure to acidic pH, followed by gradual reversing to neutral pH, reversed the HA conformational equilibrium back to that at neutral pH. The pH-modulated conformational reversibility implied the existence of a reversible fusion intermediate, in which the fusion peptide was released and distanced away from the HA base. The formation of this fusion intermediate was suggested to be before the insertion of fusion peptide into the target membrane (before the pre-hairpin intermediate). 

To sum up, smFRET imaging tackled long-standing questions regarding the timing, order, and frequency of conformational changes/transitions of HA2 during viral membrane fusion. Das et al. [29] showed how HA2 adopted different fusion-related conformations in response to acidic pH and receptors, revealing two fusion intermediates in which the fusion peptides exhibited different gestures before penetrating the target membrane (Figure 5B), consistent with previous structural observations and bulk measurements [17,131,132,133]. The first intermediate refers to the one with the fusion peptide exposed out of the hydrophobic pocket. The second intermediate corresponds to the state in which the fusion peptide releases and gets closer to the target membrane before the attachment. The findings in this research enrich our knowledge of HA-mediated membrane fusion, especially by revealing reversible and irreversible steps of HA2. Future work should focus on the dynamics of the receptor-binding subunit HA1 and the putative pre-hairpin intermediate, which was not observed due to the selection of labeling sites on HA2. Meanwhile, this study raises exciting new questions regarding the mechanism of viral membrane fusion. For instance, what are the molecular events in HA2 during switching from reversible to irreversible conformations? What are the key residues involved in this critical switching? Are there any antibodies or small molecule inhibitors that can disrupt this process? How do conformational changes in the HA1 globular head activate the fusion domain of HA2? A full understanding of influenza virus entry and identification of novel intermediates is predicted to define new drug targets.

### 3.4. smFRET Imaging of Ebola Virus Envelope Glycoprotein (GP) Revealing Roles of pH, Ca^2+^, and Receptor Binding in GP Conformations Required for Virus Entry

**Figure 6:**
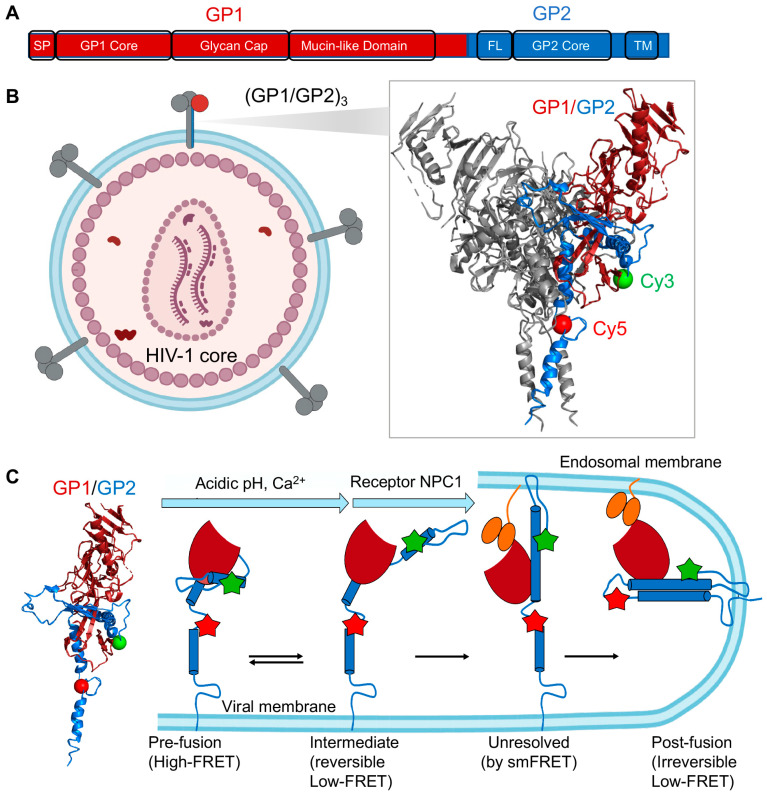
Ebola virus glycoprotein (GP)-mediated viral membrane fusion revealed by smFRET imaging. (**A**) Structural organization of Ebola virus envelope glycoprotein (GP). GP is a trimer of GP1/GP2 heterodimer. SP, signal peptide; FL, fusion loop; TM, transmembrane. (**B**) smFRET imaging of GP in the context of a lentiviral particle. A GP trimer (PDB 5JQ3) carrying a single fluorescently labeled protomer and wild-type GP trimers (gray) were incorporated into a lentiviral particle. Cy3 and Cy5 were attached to the GP2 subunit. Labeled GP protomer: GP1 in magenta; GP2 in blue; Cy3 in green; Cy5 in red. (**C**) Model of GP-mediated membrane fusion (adapted from [28]). In this model, acidic pH and Ca^2+^ facilitate GP transit from a pre-fusion conformation to an intermediate optimal for NPC1 binding, and this transition is reversible. In the intermediate conformation, the fusion loop moves from the trimer axis towards the host membrane. The binding of NPC1 then triggers at least two irreversible transitions to the post-fusion coiled-coil conformation. NPC1: Niemann-Pick C1.

In a very similar way to HA, smFRET studies of Ebola virus spike protein GP directly observed conformational dynamics and captured necessary intermediates of GP during the fusion process [28,30]. GP induces immune responses to produce antibodies against it and mediates the Ebola virus entry. GP consists of a disulfide-linked (GP1/GP2)_3_ in which GP1 facilitates engagement with cells and the cellular receptor binding, and GP2 promotes fusion of viral membrane into endosomal membranes required for virus entry (Figure 6A) [134,135,136]. Acidic pH [137], receptor Niemann-Pick C1 (NPC1) [138], and Ca^2+^ channel [139] are essential for Ebola virus GP-dependent entry. Ebola virus is first internalized into the cell through micropinocytosis, then trafficked to the late endosome where GP1 is further cleaved to remove the mucin-like domain and glycan cap [140,141,142]. The removal of these two domains in GP1 leads to the receptor-binding site exposure for NPC1 [138]. As a class I fusion protein, GP undergoes two major refolding processes, proceeding from pre-fusion to intermediate steps to post-fusion, which eventually pulls the host and viral membranes close for fusion [129]. Atomic details of GP in both pre-fusion and post-fusion (six-helix bundle) conformations have been characterized [18,19,20,21,143,144]. In the prefusion structure, the fusion loop hides in a hydrophobic cleft [21,144]. However, how conformational dynamics of GP promote Ebola virus entry has not been well characterized. For instance, what are the roles of low pH, NPC1, Ca^2+^, and glycan cap removal in Ebola virus entry? Do those factors alter conformations or dynamics of GP1 and GP2 that influence membrane fusion, and if so, how so? What are the order, the timing, and the sequence of conformational events in GP required for virus entry? Are there any irreversible or reversible steps? How can we visualize large conformational changes involving the fusion loop? From a GP conformational perspective, how can neutralizing antibodies antagonize GP?

The two most recent smFRET studies used amber-click labeling and enzymatic labeling, and touched upon the critical questions above regarding GP-mediated viral membrane fusion [28,30]. With two fluorophores labeled on GP2 (one at N-terminus, the other proximal to the fusion loop), smFRET imaging of GP on lentiviral particles permitted access to GP conformations and GP dynamics (Figure 6B,C). Das et al. [28] parallelly visualized conformational profiles of GP without mucin-like domain before and after glycan cap removal. They further monitored how the profiles responded to neutral pH, endosomal acidic pH, NPC1 receptor binding, and Ca^2+^ individually and together. Acidic pH includes irreversible exposure to endosomal acidic pH and transition exposure to endosomal acidic pH (from neutral to acidic, then back to neutral). They first observed that GP dynamically transited among multiple conformational states (reflected by high FRET, intermediate FRET, and low FRET). On the basis of observations of the high-FRET destabilization in the presence of Ca^2+^, acidic pH, glycan cap removal, and NPC1 individually or together, the authors considered high FRET as a pre-fusion state with the fusion loop sequestered. Behind the low FRET was believed to be two different conformations. The low FRET that acidic pH and Ca^2+^ shifted the conformational equilibrium towards was assigned to a pre-fusion state in which GP2 N-terminus and the fusion loop were displaced to be distant. This assignment was further supported by the restoration of conformational landscapes after re-neutralizing the endosomal acid pH. In contrast, the low FRET that the receptor NPC1 irreversibly pushed the GP2 into was implied to be the post-fusion six-helix bundle. The nature of intermediate FRET remained unknown. Collectively, conformational modulations of GP2 by endosomal pH, Ca^2+,^ NPC1, and glycan cap removal observed by smFRET imaging revealed roles of these factors in Ebola virus entry (Figure 6C): (1) low pH, Ca^2+^, and NPC1 have synergy in inducing on-path-to-fusion conformational changes in GP2, consistent with GP-mediated virus–liposome lipid mixing observed from fluorescence-based quenching assay; (2) low pH and Ca^2+^ cause reversible conformational shift of GP2 towards an intermediate, which facilitates subsequent receptor NPC1 binding; (3) GP1 with glycan cap removed renders conformational shifts of GP2 from a reversible intermediate to an irreversible post-fusion state, and thus glycan cap in GP1 likely plays a role in preventing premature activation of GP2. This study revealed two critical conformational states on the fusion pathway but inseparable in the FRET spectrum, one as a pre-fusion state, the other as the post-fusion state. Alternative labeling sites on GP2 could help the observation of clear separations among states and data interpretations.

In the second smFRET study of GP, Durham et al. [30] imaged purified protein GP ectodomain and membrane-embedded GP with mucin-like domain removed. Two FRET fluorophores were attached on GP1 and GP2 through peptide insertions associated with enzymatic labeling. They investigated the glycan gap and NPC1 binding roles in virus entry and observed similar findings to the previous smFRET study on GP2. This study provided an additional perspective (between GP1 and GP2) to observe dynamics and conformations of GP in different forms and revealed the dynamic and heterogeneous nature of GP conformations. Interestingly, the authors tested a few GP1-targeting neutralizing antibodies and showed that those antibodies had different effects in altering GP conformations. The neutralization mechanism by antibodies here was not 100% certain but seemed through inhibiting transitions to GP fusion-associated conformations. Further smFRET investigations on GP1 alone or GP1/GP2 with different labeling sites may lead to a more detailed GP antagonism mechanism by antibodies. 

The two discussed smFRET studies started to fill our knowledge gap on the conformational dynamics of Ebola glycoproteins (GPs). Several remaining questions are of interest, similar to the discussed fusion machines of influenza HA and HIV-1. How does the removal of GP1 modulate conformational changes in GP2? What is the molecular mechanism of switching between reversible and irreversible steps? Do cellular membrane compartments or other Ebola virus components besides GP play roles in Ebola viral membrane fusion? What is the molecular mechanism underlying GP antagonism by antibodies or inhibitors? Addressing such questions will facilitate a better understanding of Ebola virus entry governed by virus–host interactions.

## 4. Concluding Remarks

Virus spike–host interactions are complex and dynamic. This review covered recent fluorescence-based smFRET imaging studies on virus spike proteins of SARS-CoV-2 [31], HIV-1 [22,23,24,25,26,27,112], influenza [29], and Ebola [28,30]. These studies have connected structures and function of virus spikes in a time-resolved dimension, advanced our understanding of spike-mediated viral membrane fusion, and informed clinical interventions that target the virus entry. These studies captured intramolecular motions of virus spikes upon engaging and interacting with hosts in the milliseconds to seconds. We should note that lifetime-based FRET can achieve much higher time-resolution of microseconds to nanoseconds, which will provide additional information on dynamic aspects of virus spikes and cross the bridge to a time scale that can be studied by molecular dynamics simulations. Moreover, site-specific dye-labeling without compromising virus spikes’ functionalities, high signal-to-noise ratio, and favorable photophysical properties of organic dyes (brightness, photostability, and durability) are often technically challenging. As stable dyes and novel labeling strategies have been advanced, we can explore smFRET applications in virus spikes of other emerged and emerging viruses, such as the respiratory syncytial virus (RSV), Zika, and Dengue viruses. Besides virus spikes, smFRET imaging of other virus components that interact with hosts at the soluble protein level is feasible. However, imaging in the context of intact virus particles is currently limited to the surface proteins because components inside viral membranes are sequestered from visualization under light microscopes.

Recent smFRET studies discussed in this review have advanced our understanding of viral membrane fusion; nevertheless, many critical questions regarding this process remain elusive. One of the long-standing problems is the stoichiometry of virus glycoproteins required for successful fusion pore formation and fusion pore expansion. Technically, smFRET cannot tackle this specific question, given that the aggregate of molecules is indistinguishable at the single-molecule level. According to other non-smFRET HIV-1 Env studies in the utilization of experimental analysis and mathematical modeling, the number of HIV-1 Env glycoproteins required for virus entry varies from one digit to double-digits, and it seems to be strain-dependent [44,145,146,147]. Another long-lasting question is the nature of the putative pre-hairpin intermediate or intermediates spanning between prefusion and postfusion processes. EM work has begun to provide insights into this intermediate [148,149]. Future studies utilizing parallel smFRET, super-resolution, and cryoEM/cryoET would be most powerful in uncovering static details and dynamic aspects of virus spikes in every single step of viral membrane fusion. The achieved synergy will hold great promise for addressing these unanswered questions and will allow access to a molecular movie of viral membrane fusion.

## Data Availability

Data sharing not applicable. No new data were created or analyzed in this study. Data sharing is not applicable to this article.
